# Crop identity and memory effects on aboveground arthropods in a long‐term crop rotation experiment

**DOI:** 10.1002/ece3.5302

**Published:** 2019-05-29

**Authors:** Michael Meyer, David Ott, Philipp Götze, Heinz‐Josef Koch, Christoph Scherber

**Affiliations:** ^1^ Institute of Landscape Ecology University of Münster Münster Germany; ^2^ Agronomy Department Institute of Sugar Beet Research Göttingen Germany

**Keywords:** agriculture, agroecosystem, arthropods, biodiversity, community composition, crop diversity, temporal diversification

## Abstract

Agricultural landscapes are globally dominated by monocultures under intensive management. This is one of the main reasons for biodiversity loss and insect population decline in many regions all over the world. Agroecosystem biodiversity in these areas can be enhanced by cropping system diversification, such as crop rotations. Yet, long‐term studies on effects of crop rotations on aboveground agrobiodiversity are lacking. We set up a 10‐year long‐term crop rotation experiment in Central Germany and monitored the temporal dynamics of aboveground arthropods over a full cultivation period to investigate influence of current and preceding crop identity and cropping system diversification on activity density, species richness, and community structure. We found that species composition was strongly influenced by currently grown crop although effect on arthropods varied between species groups. Especially, winter oilseed rape strongly affects arthropod community structure. Interestingly, we were also able to show an influence of the preceding crops, indicating an ecological memory effect in the aboveground arthropod community. Our results show that crop identity of both currently and previously grown crops in crop rotations may lead to an increase in arthropod activity density and changes in species composition. Diversified crop rotations including appropriate crops can be an easily implemented tool to increase arthropod biodiversity and biomass at large spatial and temporal scales, particularly in areas dominated by a single crop (e.g., wheat, maize). Our results may help to design optimized crop rotations for large‐scale enhancement of insect biodiversity in agroecosystems.

## 1 INTRODUCTION

Long‐term crop monocultures are still common in many parts of the world, yet evidence is mounting that more diverse cropping systems are needed to reconcile agricultural productivity and environmental sustainability (Bennett, Bending, Chandler, Hilton, & Mills, 2012; Liebman & Schulte, 2015). Agroecosystems can be diversified in two major ways: spatial diversification (e.g., intercropping or field margin management) and temporal diversification (e.g., crop rotations), or combinations of both. In Europe, the three‐field system is an ancient example of a crop rotational system that has been used since the Middle Ages, showing that people already were aware of the benefits of cropping sequences compared to mono‐cropping (Brankatschk & Finkbeiner, 2015; Lochner & Breker, 2011). Today's crop rotations (including catch or cover crops) are widely used to inhibit the development of deleterious organisms by breaking the life cycles of pest taxa such as bacteria, fungi, nematodes, or insects (Bennett et al., 2012; Brankatschk & Finkbeiner, 2015; Dias, Dukes, & Antunes, 2015; Esser, Milosavljević, & Crowder, 2015; Robinson & Sutherland, 2002; Tiemann, Grandy, Atkinson, Marin‐Spiotta, & McDaniel, 2015) or to avoid self‐inhibition due to autotoxicity of the main crop (Bennett et al., 2012). Crop rotations may lead to a decrease in pesticide or fertilizer use and can therefore increase economic benefit (Brankatschk & Finkbeiner, 2015; Struik & Bonciarelli, 1997).

The preceding crop (e.g., oilseed rape) may directly or indirectly affect organisms occurring in the main crop (e.g., wheat). Such effects are an example of an ecological memory effect that has frequently been reported for soil systems (Bengtsson et al., 2003; Lapsansky, Milroy, Andales, & Vivanco, 2016; Ogle et al., 2015; Peterson, 2002). From a systems perspective, ecological memory refers to the composition of species, interactions and structures that make ecosystem reorganization possible (Bengtsson et al., 2003), whereby past modifications of this composition determine the degree to which a current ecological process is shaped (Ogle et al., 2015; Peterson, 2002). Lapsansky et al. (2016) followed the ecological memory perspective and proposed soil memory in the context of agroecosystems, summarizing the association between host plants of specific crops, symbionts, and pathogens (Lapsansky et al., 2016).

Up to now, studies focused primarily on effects of crop rotations on aspects of soil health, such as soil texture, physicochemical characteristics, soil microbial biomass, or microbial composition. Yet, there is increasing awareness that crop rotations also affect organisms above ground, but only few studies investigated if and how temporal diversification would also affect aboveground taxa and community structure. While crop rotations are frequently employed, for example, in Central European farmland, the exact sequences of crops grown are often unknown due to limited access to farm inventory data. Further, remote‐sensing approaches to derive large‐scale crop cover estimates are still in development (Dahal, Wylie, & Howard, 2018).

**Figure 1:**
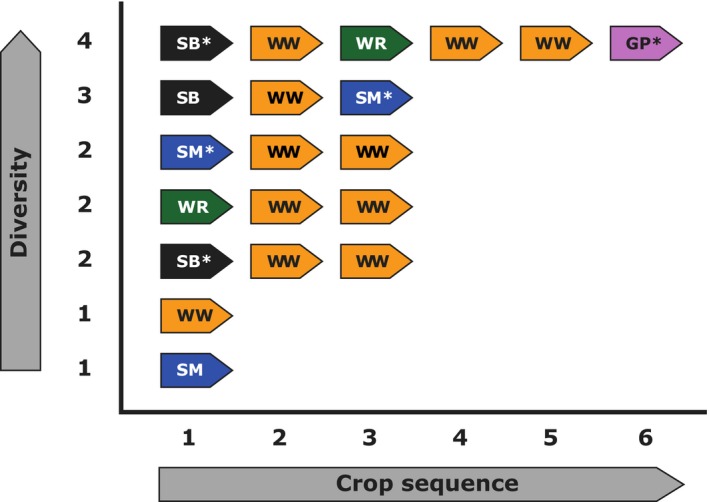
Overview of crop rotations: *Crop sequence* indicates the realized sequence of crop species per rotation, while *diversity* indicates the number of crop species in a particular rotation. Crops included sugar beet (SB, black color), winter wheat (WW, yellow color), silage maize (SM, blue color), winter oilseed rape (WR, green color), and grain pea (GP, pink color). Asterisks indicate if management regime included cover plants for a crop species, that is, phacelia (*Phacelia tanacetifolia* Benth.) for GP and mustard (*Sinapsis arvensis* L.) for SB and SM

**Figure 2:**
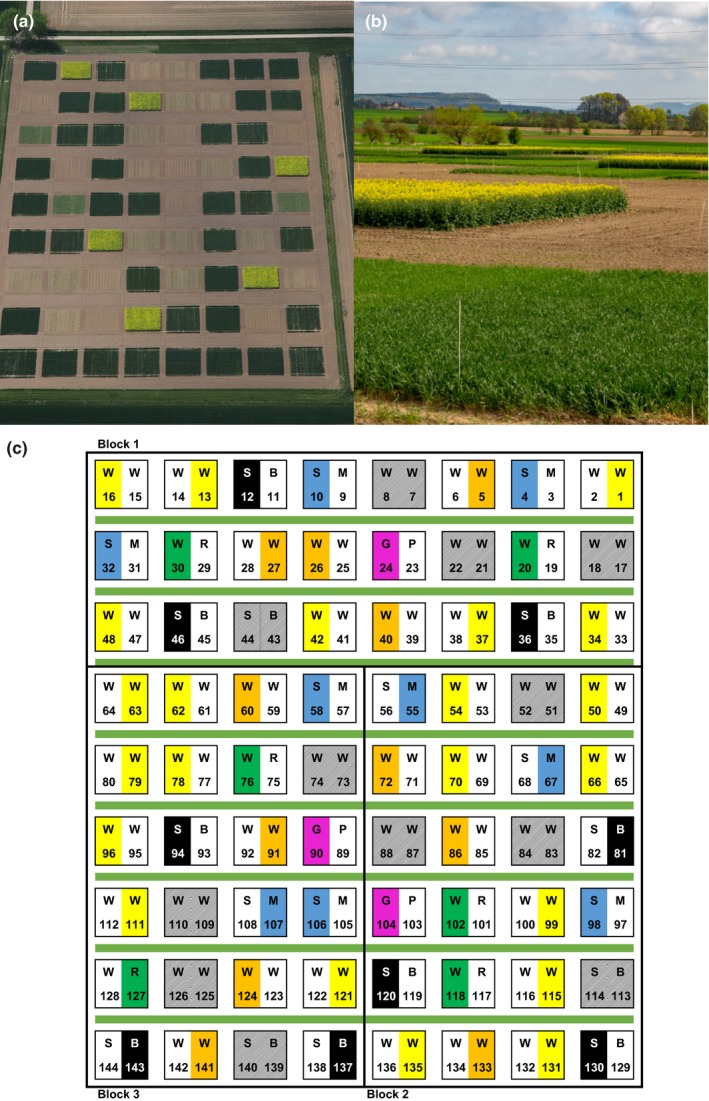
Details on the layout of the crop rotation experiment. (a) Aerial photograph showing the spatial arrangement of plots (taken by Andreas Krukemeyer, May 2011); (b) close‐up view of plots; (c) field plan of the experiment; the whole field consisted of 72 plots of which 12 were not used in this study (shaded). Plots that were included in the experiments are marked with colors according to currently grown crop as described in Figure 1 (GP, grain pea in pink; SB, sugar beet in black; SM, silage maize in blue; WR, winter oilseed rape in green; WW, winter wheat in yellow). WW was sown at different times (bright yellow: WW sown in September, orange: WW sown in October). We used only the right‐ or the left‐hand side of a plot (drawn at random). Numbers refer to plot numbers. Green lines indicate grass strips between plots. All plots were surrounded by a bare edge with a width of approximately 2.6 m. Figure 2c covers with the aerial photograph but was taken in 2011; therefore, crops in the photograph (e.g., oilseed rape) do not match with crops grown 2016 as described in Figure 2c

Here, we use a 10‐year crop rotation experiment (Figures 1 and 2) to study long‐term effects of crop rotation and crop identity on temporal dynamics of aboveground arthropod taxa. The crop rotation experiment consists of nine different crop rotations; of these, we used seven rotations, representing the most important intensively managed arable crop rotations present in Central Germany. Rotations spanned a gradient in diversity from one (continuous mono‐cropping) to four (four main crops), subsequently termed “temporal crop diversity.” All crops in each rotation were grown each year on separate plots measuring c. 230 m^2^ with three replicate blocks, giving a total of *N* = 72 plots whereof we used *N* = 60 plots.

We test the following hypotheses:

1. Crop species identity affects arthropod activity density, species richness, or community composition.
2. Temporal crop diversity has a positive effect on arthropod activity density and species richness.
3. The preceding crop(s) grown in previous years will influence current arthropod communities (memory effect), depending on life cycle and feeding behavior of species.

## 2 MATERIALS AND METHODS

### 2.1 Experimental design

We sampled invertebrates in a long‐term crop rotation experiment in Central Germany. The experiment was established in 2006 near the village of Harste, about 6 km north of the city of Göttingen (51°36′23″N, 9°51′56″E). The soil is a stagnic Luvisol derived from Loess with a silt content of 86% and a clay content of 12% (Institute of Sugar Beet Research, unpublished data).

The design was a randomized complete blocks design with three blocks, and nine rotations (Figures 1 and 2) comprising *N* = 72 plots of whom seven rotations and *N* = 60 plots were used. These contained the crop species sugar beet (SB; *Beta vulgaris* L.), winter wheat (WW; *Triticum aestivum* L.), silage maize (SM; *Zea mays* L.), winter oilseed rape (WR; *Brassica napus* L.), and grain pea (GP; *Pisum sativum* L.) either in sequence or under continuous cropping (Figure 1). Additionally, the catch crops mustard (*Sinapis alba* L., MU) and phacelia (*Phacelia tanacetifolia* L., PH) were cultivated prior to SB, SM (catch crop MU), and GP (catch crop PH). Plots had a size of 16.2 by 14.0 m and were surrounded by bare soil (c. 2.6 m width) or small grass strips (Figure 2). The plots were managed according to good farming practice with crop‐specific soil cultivation, pesticide application, and fertilization. Only WW received a single insecticide application, which we consider negligible in the context of this study.

### 2.2 Assessment of management intensity

To further characterize management intensity, we defined the following variables: (a) soil cultivation, that is, the sum of all ploughing, harrowing, and sowing events since the harvest of the last main crop; (b) pesticide application, the sum of herbicide, fungicides, molluscicide, insecticide, and rodenticide applications; (c) fertilization, the application events of N, P, K, S (or combinations), and growth regulators.

### 2.3 Arthropod sampling

Aboveground arthropods were sampled in 2016, between spring sowing (April) and the onset of summer harvest of WW, GP, and WR (July). On each of the 60 plots, we installed one funnel trap (modified pitfall trap; 10 cm diameter, with a rain shelter). Traps were kept active for 2 weeks at four times during the study period, resulting in 54 trapping days (21 April to 4 May, 19 May to 1 June, 16 June to 30 June, and 4 July to 21 July). The trapping liquid was propylene glycol (IUPAC name: propane‐1,2‐diol; dilution 1:2, Karl Roth GmbH & Co. KG). Samples were transferred to 70% (w/v) ethanol (Waldeck GmbH & Co. KG) and stored in a refrigerator at 4°C until further identification to species level. Ground beetle larvae were discarded, as pitfall traps are not an adequate sampling method for this group. Specimen that could not be identified to species level (i.e., juvenile and subadult spiders and juvenile isopods and diplopods) or damaged individuals were identified to genus or family level, excluding double counts. Our sampling included both ground‐dwelling spiders and spiders that build webs at the soil surface (termed “web spiders” hereafter).

### 2.4 Statistical analysis

Data were analyzed using the statistical software package R, version 3.5.1; (R Core Team, 2018). Explanatory variables were the crop grown per plot (a factor with five levels: GP, SB, SM, WR, and WW) in the years 2016, 2015, and 2014, and the number of unique main crop species within a rotation (numeric; “temporal crop diversity”; ranging from one (continuous cropping) to four species, Figure 1). We did these analyses (a) for all crops included in the rotation and (b) for plots only grown with SB, SM, and WW. To analyze arthropod activity density data, we summed arthropod individual numbers per taxon (carabids, web spiders, and iso‐/diplopods) for each plot (*N* = 60) and sampling date. To analyze species richness data, we calculated the numbers equivalent of Shannon's diversity index H for each plot and sampling date (exp(H)) (Jost, 2007) using the R package “vegan,” version 2.5.2 (Oksanen et al., 2018).

To test for general patterns averaged over time, we analyzed the effects of crop identity and cropping system diversity on the annual sum of activity density and species richness (exp(H)) per plot. For these analyses, we used generalized linear mixed‐effects models fit by penalized quasi‐likelihood with blocks as random effects to account for spatial non‐independence between plots. Equivalent models fit by generalized least squares, with *x* and *y* coordinates of the plots entered as spatial correlation structures, yielded identical results. To assess effects of single crops, we employed multiple comparisons in R package “multcomp” version 1.4‐8 (Hothorn, Bretz, & Westfall, 2008).

To assess the temporal dynamics of arthropods within a year, we used generalized additive mixed models (GAMM) in R package “mgcv” (Wood, 2017) with time as a smooth term with factor interactions. Response distributions (and hence normality of errors) were assessed using the “fitdistrplus” package in R version 1.0‐9 (Delignette‐Muller & Dutang, 2015). Since activity density data were positive counts, we used Poisson or Tweedie distributions with varying index parameter. The index parameter was estimated using the tw() function in a basic GAM without random effects. Arthropod activity density and species richness were modeled as a function of sampling date using thin plate splines, calculating separate smooths for crop grown in 2016, 2015, and 2014. The model formula contained: (a) a factor smooth interaction term: s(time, by = crop*_i_*, bs = “ts”), where *I* = {2016; 2015; 2014}; and (b) a random effect for each plot: random = list(plotcode ~ 1). The degree of smoothness of the smooth terms was defined by adding the argument “select = T” to the model call; this allows to impose penalties on the smoothing parameter of each term, so that terms can also be completely become penalized out of the model (Wood, 2017, p. 315). An alternative nonlinear model specification using nonlinear mixed Gaussian regression models (SSGauss) did not converge. Including location of the plots as a spatial smooth term s(*x*,*y*) did not change the outcome of the model; therefore, position of the plot in the field was ignored.

Using the same GAMM approach, we modeled arthropod activity density and species richness as a function of cropping system diversity (“temporal crop diversity”) and sampling date.

Multivariate analyses of arthropod community composition were performed using principal components analysis (PCA) in CANOCO 5 (Microcomputer Power, ter Braak & Šmilauer, 2012). Species data were log‐transformed before analyses to avoid distortions caused by highly abundant species.

## 3 RESULTS

### 3.1 Major arthropod taxa and dominant species

In total, we sampled 8,313 individuals of ground beetles (46 species), 6,341 web spiders and harvestmen (60 species), 741 diplopods (eight species), and four isopod individuals (three species). In subsequent analyses, we pooled diplopods and isopods. Dominant ground beetle species were *Pterostichus melanarius* (Illiger), *Trechus quadristriatus* Schrank, *Amara similata* (Gyllenhaal), and *Nebria salina* Fairmaire. These constituted about 80% of all individuals in the beetle community. Spider communities were dominated by *Collinsia inerrans* O. P. Cambridge, *Oedothorax apicatus* (Blackwall), *Erigone atra* Blackwall, and *Porrhomma microphthalmum* (O. P. Cambridge), which constituted about 60% of individuals. The most dominant millipede species were *Polydesmus inconstans* Latzel and *Unciger foetidus* (C. L. Koch). Across all taxonomic groups, the identified species were ubiquitous and common for agricultural landscapes.

### 3.2 Effects of crop identity on arthropod activity density and species richness

**Figure 3:**
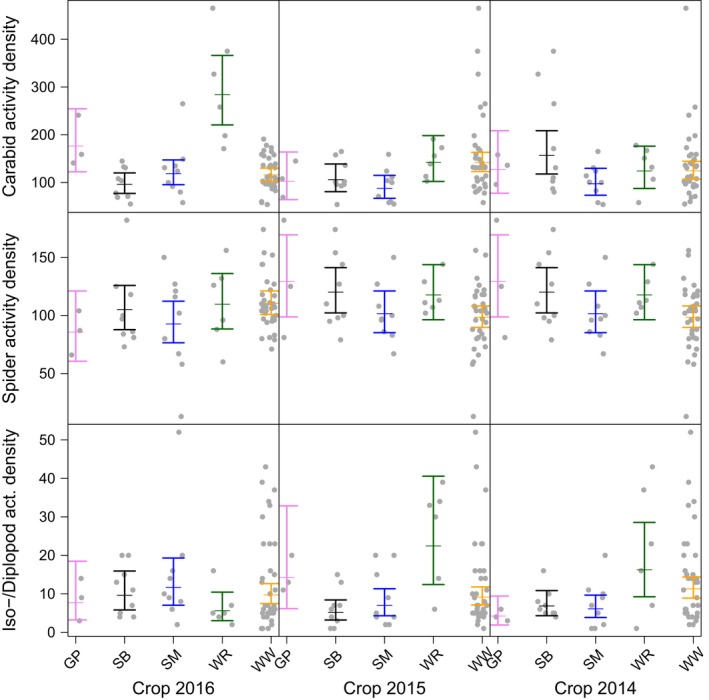
Effect of current crop (grown in year 2016, panels in left column) and preceding crops (grown in years 2015 and 2014, panels in middle and right column, respectively) on activity density (i.e., cumulative number of individuals across species per taxon in pitfall traps) of carabid beetles, web spiders, and isopods and diplopods combined (rows from top to bottom). All individual counts are based on data collected in 2016. Colors indicate individual crop species (GP, grain pea in pink; SB, sugar beet in black; SM, silage maize in blue; WR, winter oilseed rape in green; WW, winter wheat in yellow). Dots show the samples; bars show 95% confidence intervals around the mean (estimated from generalized linear mixed‐effects models)

**Figure 4:**
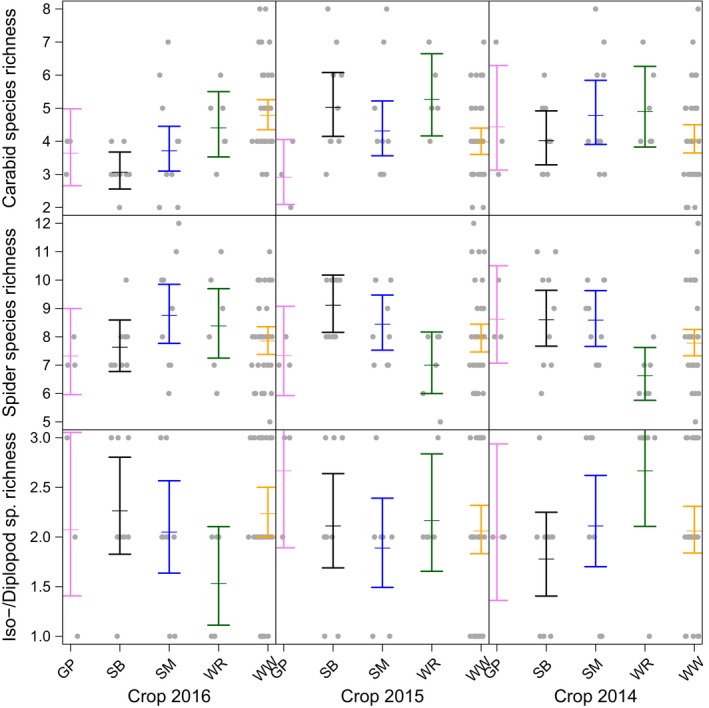
Effect of current crop (grown in year 2016, panels in left column) and preceding crops (grown in years 2015 and 2014, panels in middle and right column, respectively) on species richness (numbers equivalents, i.e., the exponential of Shannon diversity) of carabid beetles, spiders, and isopods and diplopods combined (rows from top to bottom). Colors indicate individual crop species (GP, grain pea in pink; SB, sugar beet in black; SM, silage maize in blue; WR, winter oilseed rape in green; WW, winter wheat in yellow). Dots show the samples; bars show the 95% confidence intervals around the mean (estimated from generalized linear mixed‐effects models)

There were significant effects of current crop (2016) and preceding crops (2015 and 2014) on activity densities of carabids and iso‐/diplopods, with similar (but nonsignificant) patterns in web spiders (Table 1 and Figure 3). The species richness of carabids and web spiders was also significantly affected by crop identity in the current and even the previous 2 years (Table 1 and Figure 4), but these effects were much weaker than effects on activity density.

**Table 1:** Type II analysis of deviance tables for GLMMs on effects of crop type and crop diversity on activity density and species richness (exponential Shannon diversity). Note that different models were run for crop identity versus temporal crop diversity

Response variable	Species	Predictor	*χ* ^2^	*df*	Pr(<*χ* ^2^)
Activity density	Carabids	Crop 2016	**84.36**	**4**	**<2.2e‐16**
		Crop 2015	**9.67**	**4**	**0.046**
		Crop 2014	6.17	4	0.187
		Temp. Crop diversity	2.40	1	0.121
		Temp. div (SB)	0.08	1	0.779
		Temp. div (SM)	2.91	1	0.088
		Temp. div (WW)	1.51	1	0.219
	Web spiders	Crop 2016	4.41	4	0.354
		Crop 2015	8.40	4	0.078
		Crop 2014	4.08	4	0.395
		Temp. Crop diversity	1.31	1	0.253
		Temp. div (SB)	3.07	1	0.080
		Temp. div (SM)	0.97	1	0.325
		Temp. div (WW)	1.28	1	0.256
	Iso‐/Diplopods	Crop 2016	3.24	4	0.518
		Crop 2015	**14.43**	**4**	**0.006**
		Crop 2014	**12.61**	**4**	**0.013**
		Temp. Crop diversity	0.53	1	0.468
		Temp. div (SB)	**5.27**	**1**	**0.022**
		Temp. div (SM)	0.01	1	0.912
		Temp. div (WW)	1.94	1	0.164
Sp. richness (eH)	Carabids	Crop 2016	**19.39**	**4**	**0.001**
		Crop 2015	**12.50**	**4**	**0.014**
		Crop 2014	4.14	4	0.387
		Temp. Crop diversity	0.00	1	0.945
		Temp. div (SB)	0.00	1	1.000
		Temp. div (SM)	0.00	1	1.000
		Temp. div (WW)	1.91	1	0.168
	Web spiders	Crop 2016	5.11	4	0.276
		Crop 2015	**9.96**	**4**	**0.041**
		Crop 2014	**10.95**	**4**	**0.027**
		Temp. Crop diversity	0.22	1	0.639
		Temp. div (SB)	0.19	1	0.660
		Temp. div (SM)	0.05	1	0.830
		Temp. div (WW)	0.13	1	0.724
	Iso‐/Diplopods	Crop 2016	5.59	4	0.232
		Crop 2015	2.89	4	0.576
		Crop 2014	6.32	4	0.177
		Temp. Crop diversity	0.45	1	0.503
		Temp. div (SB)	2.62	1	0.106
		Temp. div (SM)	0.00	1	1.000
		Temp. div (WW)	0.18	1	0.671

Bold values indicate a significant effect of the predictor variable on activity density or species richness of the particular group.

When looking at individual crops (Tables S1, S2, S3), both WR and GP had strongly positive effects on both activity density (Tables S1 and S2) and species richness (Table S3) of individual taxa. For example, plots grown with WR had more than 280 individuals of carabid beetles in 2016, while only about 100 individuals were found in SB. The same was true for the preceding crop: We found c. 140 carabid individuals in plots that had been grown with WR in the year before (crop*_t_*_−1_, 2015), while only c. 90 individuals were found when a plot had been grown with SB. Isopod and diplopod activity densities were highest in plots grown with SM 2016 (Table S1), but these differences were not significant (Table 1).

### 3.3 Temporal dynamics of arthropods

**Figure 5:**
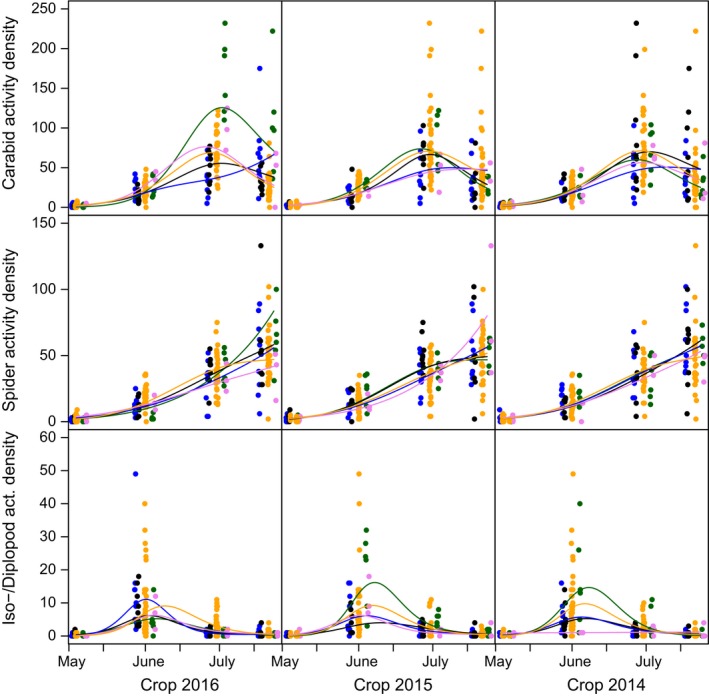
Temporal dynamics of activity density (i.e., cumulative number of individuals across species per taxon in pitfall traps) of carabid beetles (panels in uppermost row), web spiders (panels in middle row), and isopods and diplopods combined (panels in lowermost row) in 2016 as a function of crop species identity. Model predictions are shown for current crop (grown in year 2016, panels in left column) and preceding crops (grown in years 2015 and 2014, panels in middle and right column, respectively). Colors correspond to grain pea (GP, pink), sugar beet (SB, black), silage maize (SM, blue), winter oilseed rape (WR, green), and winter wheat (WW, yellow)

**Figure 6:**
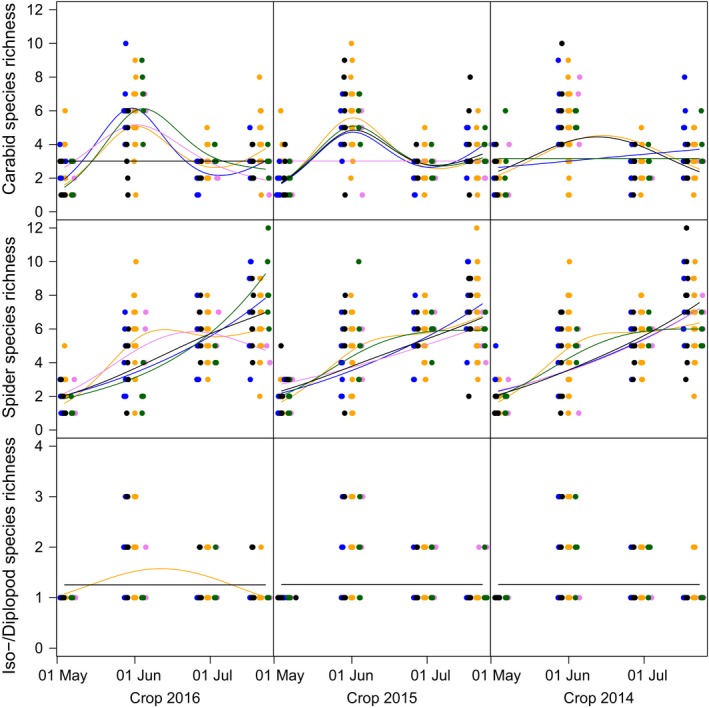
Species richness (numbers equivalents, i.e., the exponential of Shannon diversity) of carabid beetles (panels in uppermost row), spiders (panels in middle row), and isopods and diplopods combined (panels in lowermost row) during sampling season 2016 as a function of crop species identity. All individual counts are based on data collected in 2016. Model predictions are shown for current crop (grown in year 2016, panels in left column) and preceding crops (grown in years 2015 and 2014, panels in middle and right column, respectively). Colors correspond to grain pea (GP, pink), sugar beet (SB, black), silage maize (SM, blue), winter oilseed rape (WR, green), and winter wheat (WW, yellow)

Both activity densities and (to a lesser extent) species richness of arthropods showed strong temporal dynamics that were modified significantly by crop identity in the current and previous years (Figures 5 and 6). Isopods and diplopods reached highest activity densities in early spring, followed by ground beetles in late spring, whereas web spiders reached highest individual numbers in summer. Crop identity effects were generally strongest for the current crop and dampened if crops from previous years were considered. Interestingly, for iso‐/diplopods, the crops grown at *t*_−1_ and *t*_−2_ showed stronger effects on activity density than the current crop (Figure 5). Model predictions are shown for minimum adaequate GAM‐models. Straight lines of model fits indicate non‐significant effects. All other effects were signifcant.

### 3.4 Effects of temporal crop diversity on arthropod activity density and arthropod species richness

**Figure 7:**
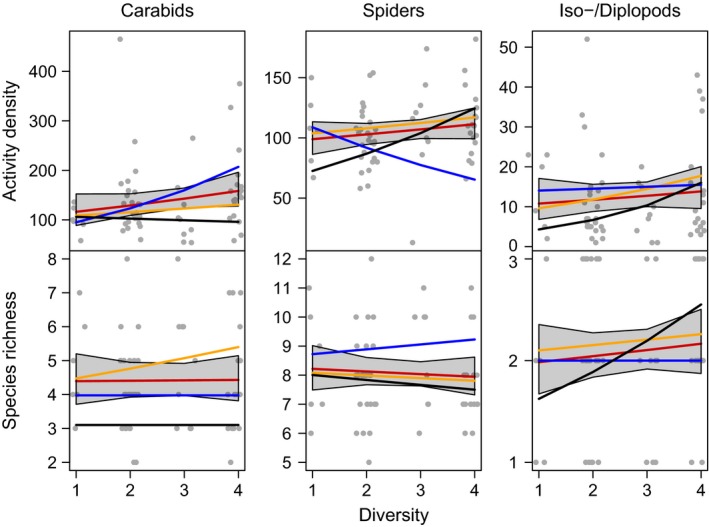
Effects of temporal cropping system diversity (i.e., richness in crop species in a crop rotation) on activity density (i.e., cumulative number of individuals across species per taxon in pitfall traps) and species richness (numbers equivalents, i.e., the exponential of Shannon diversity) of carabid beetles (panels in left column), web spiders (panels in middle column), and isopods and diplopods combined (panels in right column). Red lines show model predictions including all crops with 95% confidence bands, yellow lines show model outcome for plots grown with WW only, black for SB, and blue for SM

**Figure 8:**
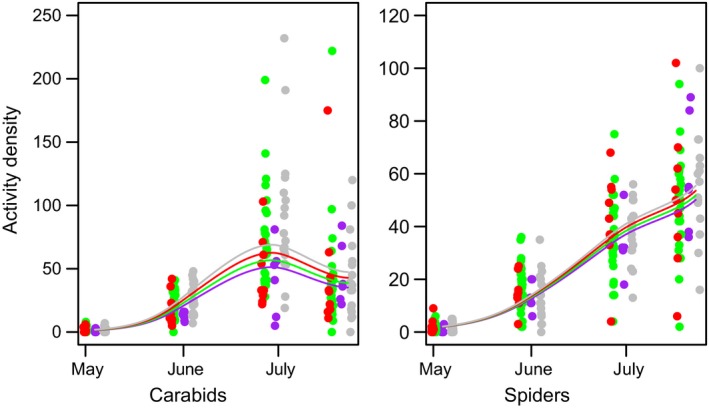
Effects of cropping system diversity on temporal dynamics of activity density (i.e., cumulative number of individuals across species per taxon in pitfall traps, including all plots) in 2016. Colors indicate cropping system diversity: continuous cropping of one crop (purple), 2 crops (green), 3 crops (red), and 4 crops (gray)

**Figure 9:**
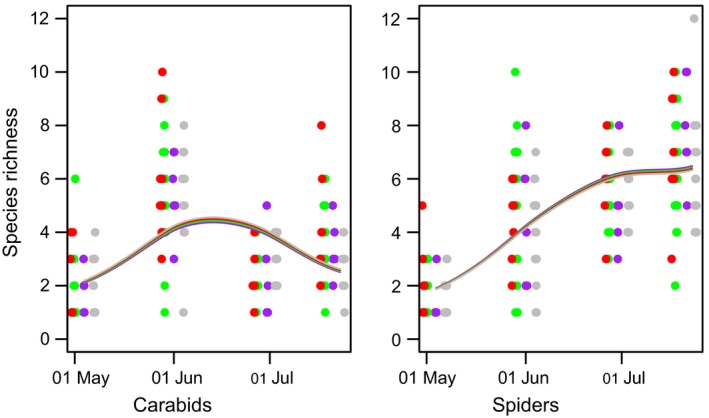
Species richness (numbers equivalents, i.e., the exponential of Shannon diversity, including all plots) over the course of the growing season 2016 as a function of temporal cropping system diversity. Colors indicate cropping system diversity: continuous cropping of one crop (purple), 2 crops (green), 3 crops (red), and 4 crops (gray)

All three arthropod taxa showed higher activity densities with increasing temporal crop diversity, although this trend was not significant and showed different patterns depending on the crop included in the model (Table 1 and Figure 7). We conducted the analyses for all crops and additionally restricted to individual crops (SB, SM, and WW) to disentangle effects of individual crops and temporal crop diversity (Figure 1). When comparing models on crop identity with models on temporal crop diversity, crop identity models had pseudo‐*R* ^2^ values of c. 0.92 (carabids), 0.39 (spiders), and 0.43 (iso‐/diplopods), while temporal crop diversity models had lower pseudo‐*R* ^2^ values of c. 0.6, 0.15, and 0.8, indicating lower explanatory power of temporal crop diversity than crop identity. Models on temporal dynamics of activity density (Figure 8) and species richness (Figure 9) as a function of temporal crop diversity showed only minor effects of temporal crop diversity.

### 3.5 Arthropod community composition

**Figure 10:**
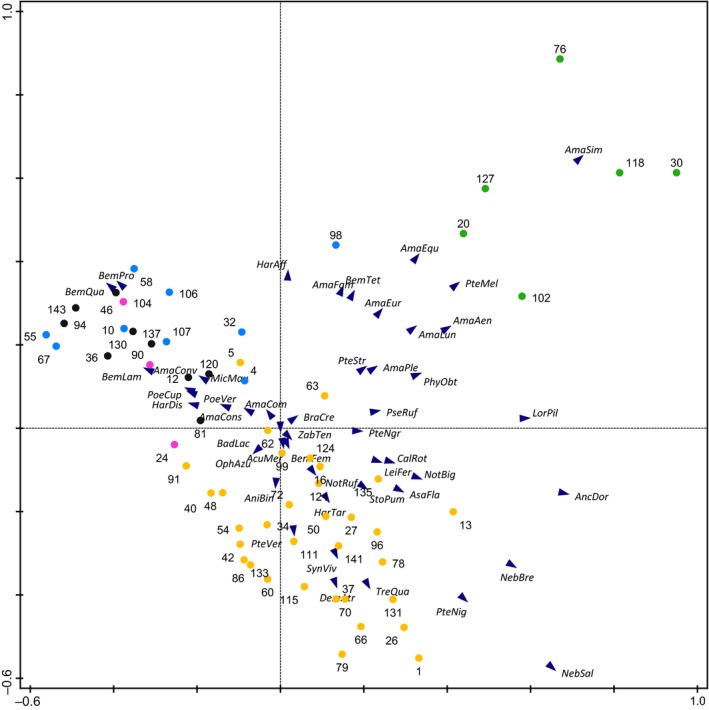
Species composition of carabid beetles in PCA space. Plots’ labels show by current crop in 2016. Colors indicate individual crop species (GP, grain pea in pink; SB, sugar beet in black; SM, silage maize in blue; WR, winter oilseed rape in green; WW, winter wheat in yellow). Numbers represent plot number and abbreviations represent species. (Eigenvalues: 0.241, 0.152, 0.082, 0.075, for abbreviations see data at Dryad)

Community composition in carabids was strongly affected by the current crop grown in 2016 (Figure 10): Plots grown with WR had strongly different community composition, mainly influenced by species of the Genus *Amara* that feed both on springtails and also on seeds, and other omnivorous species as *Pterostichus melanarius* (Illiger) but also by carnivorous species such as *Loricera pilicornis* (Fabricius). Plots grown with WW showed a species composition characterized by both phytophagous and carnivorous beetle species. Species composition in plots grown with GP, SM, and SB was more homogenous and dominated by three species of the genus *Bembidion*.

**Figure 11:**
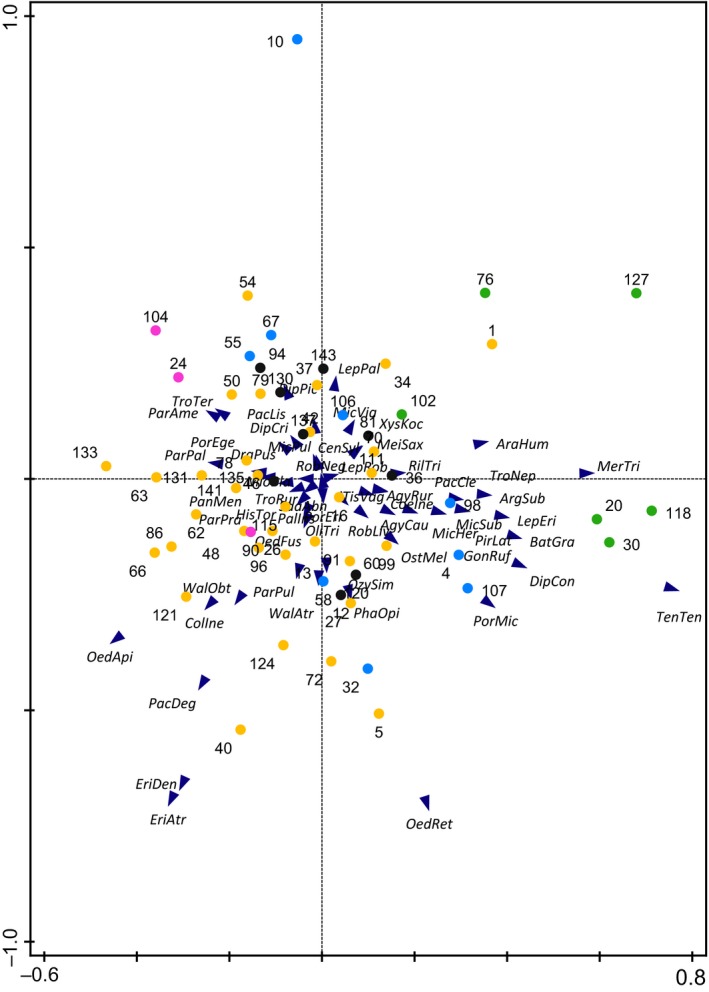
Species composition of spiders in PCA space. Plots’ labels show current crop in 2016. Colors indicate individual crop species (GP, grain pea in pink; SB, sugar beet in black; SM, silage maize in blue; WR, winter oilseed rape in green; WW, winter wheat in yellow). Numbers represent plot numbers; abbreviations represent species. (Eigenvalues: 0.165, 0.153, 0.124, 0.084, for abbreviations see data at Dryad)

In web spiders, only plots grown with WR differed in species composition compared to plots grown with other crops (Figure 11). WR plots showed more species that build sheet webs in the vegetation, for example, *Bathyphantes parvulus* (Westring) and *Tenuiphantes tenuis* (Blackwall). Ground dwellers, such as wolf spiders (Lycosidae) and various Erigoninae spiders that build webs at the soil surface, occurred on all plots without any preference for a certain crop. For isopods and diplopods, no clear pattern was found due to low species numbers.

## 4 DISCUSSION

The analyses presented here clearly show that crop species identity affects arthropods in intensive agricultural production systems, supporting hypotheses one. Temporal crop diversity, however (hypothesis two), had no consistent effects on arthropods: although some species groups had higher activity densities in more diverse rotations, some groups showed the opposite pattern. However, and quite surprisingly, we found that the preceding crops grown up to 2 years ago still apparently affected arthropods collected in a given year, supporting hypothesis three. Such a “memory effect” of preceding crops has (to our knowledge) never been reported so far for aboveground species. However, legacy effects of soil fauna and microbiota and indirect effects on aboveground species have been previously reported (Kostenko, van de Voorde, Mulder, van der Putten, & Martijn Bezemer, 2012).

Effects of individual crop species on ground‐dwelling invertebrates have frequently been reported, especially for carabid beetles (Eyre, Luff, Atlihan, & Leifert, 2012; Eyre, Luff, & Leifert, 2013; Eyre, McMillan, & Critchley, 2016; O'Rourke, Liebman, & Rice, 2008). Crop identity can affect invertebrates through two major pathways: (a) direct effects, such as differences in crop density (sowing rate), phenology (sowing date), or management intensity combined with differences in crop‐specific traits, and (b) indirect effects via the decomposer subsystem.

The probability of catching a particular surface‐dwelling taxon in a pitfall trap will likely be affected by vegetation structure (Koivula, Kotze, Hiisivuori, & Rita, 2003) or sowing density or date. In ground‐dwelling arthropods, the vegetation structure directly at soil surface (stem density or litter laying on the ground) affects activity density. At the beginning of the season, activity density was comparatively low in all crops due to the cold weather conditions but differed when temperature increased. Later in the season, structurally rich crops such as WW, GP, and WR showed higher activity densities in ground beetles compared to SB and SM. These observations are consistent with findings of O'Rourke et al. (2008), who showed that a structurally rich crop that creates a canopy early in the season increases ground beetle activity density and even diversity (O'Rourke et al., 2008). Plots grown with SM in the current year and with WR in the previous year showed high activity densities of decomposers, such as millipedes and isopods that profit from plant residuals left in the field or leaves and petals falling down from the plants. Web spiders were less influenced by currently grown crop. Many of the species we found are hunting on the soil surface or build sheet webs on the ground and are very small and might therefore not be influenced by vegetation structure.

**Figure 12:**
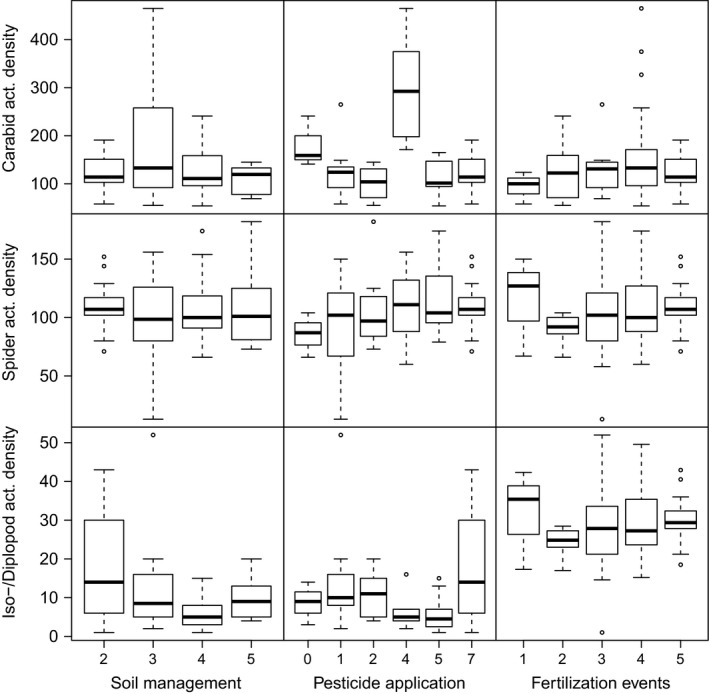
Effects of soil management, pesticide application, and fertilization events on activity density (i.e., cumulative number of individuals across species per taxon in pitfall traps) of carabid beetles, spiders, and isopods and diplopods combined (rows from top to bottom)

Arthropod activity density could also have been affected by crop‐specific management practices (Eyre et al., 2012; Purvis, Fadl, & Bolger, 2001). Yet, neither number of fertilization events nor pesticide application or soil management per se had a clear influence on activity density of ground beetles, spiders, and iso‐ and diplopods—because the number of management events was crop‐specific and can therefore not be disentangled from crop identity in our experiment (Figure 12 and Table 2). For example, WR was the only crop in our experiment that received pesticides four times per year. Overall, cropping systems are always somewhat “artificial” habitats with crop species‐specific management; disentangling crop identity from management would require an explicit manipulation of management intensity. This is also true for (potential) effects of cover crops that were also specific to particular crops. Other experiments (explicitly manipulating cover crop identity) may be more suitable to test for these effects.

**Table 2:** Effects of management practice on activity density (analysis of deviance, type II, based on GLMMs)

Activity density	Predictor	*χ* ^2^	*df*	Pr(<*χ* ^2^)
Carabids	Soil management	**8.38**	**3**	**0.039**
	Pesticide application	**86.03**	**5**	**<2.2E‐16**
	Fertilization events	5.56	4	0.235
Spiders	Soil management	1.56	3	0.669
	Pesticide application	4.74	5	0.449
	Fertilization events	3.43	4	0.488
Iso‐/Diplopods	Soil management	**12.24**	**3**	**0.007**
	Pesticide application	**16.50**	**5**	**0.006**
	Fertilization events	**19.65**	**4**	**<0.001**

Bold values indicate a significant effect of the predictor variable on activity density or species richness of the particular group.

**Figure 13:**
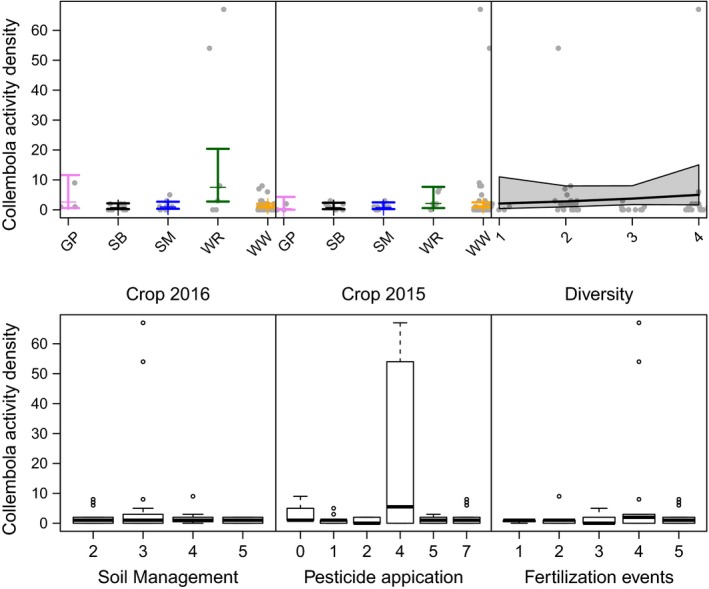
Effects on the cumulative activity density of the mesofauna (i.e., cumulative number of individuals across all species of collembola extracted from soil cores). Upper row (panels left to right): effect of current crop (grown in year 2016), preceding crops (grown in year 2015), and crop species diversity in a rotation. Colors indicate individual crop species (GP, grain pea in pink; SB, sugar beet in black; SM, silage maize in blue; WR, winter oilseed rape in green; WW, winter wheat in yellow). Lower row, panels left to right: effects of soil management, pesticide application, and fertilization events. Lines show model predictions with 95% confidence bands

Community composition may also be mediated indirectly via the decomposer subsystem. For example, high collembolan densities may lead to an increase in ground‐dwelling predators (Birkhofer, Wise, & Scheu, 2008). Indeed, additional analyses (Figure 13 and Table 3) showed that collembolans reached higher densities in plots grown with WR in 2016 and 2015 (although this signal dampened 1 year after WR growth) which may partly explain higher densities of carabid beetles. However, mesofauna was unaffected by temporal crop diversity, and we also detected no clear effects of management or pesticide application on mesofauna.

**Table 3:** Effects of crop identity, crop temporal diversity, and management practice on activity density (analysis of deviance, type II, based on GLMMs)

Collembola activity density	*χ* ^2^	*df*	Pr(<*χ* ^2^)
Crop2016	**74.04**	**4**	**3.18E‐12**
Crop2015	2.50	4	0.646
Crop diversity	0.56	1	0.444
Soil management	**7.88**	**3**	**0.048**
Pesticide application	**75.53**	**5**	**7.22E‐12**
Fertilization events	5.94	4	0.204

Bold values indicate a significant effect of the predictor variable on activity density or species richness of the particular group.

Overall, crop identity had a stronger effect on arthropod community composition than temporal crop diversity, especially when looking at species richness. The influence of crop identity and crops grown in previous years on community composition will of course also strongly depend on life history, life span, trophic position, and mobility of the investigated species. For example, web spiders have a lifespan of usually less than 1 year and many species are very mobile because of ballooning (Heimer & Nentwig, 1991). Thus, species composition was rather similar between all crops except WR because this crop provides a more complex vegetation structure for building webs in the vegetation (and higher humidity; personal observation). Communities of web spiders are therefore “reshuffled” every year, depending on crop identity and microclimatic preferences of individual species.

Diplopods can live up to several years (Voigtländer, 1992) and are less mobile than spiders in general. Thus, it is likely that they can show multi‐annual memory effects, as long as shallow ploughing (in our study only 10 cm depth) had no effect.

For ground beetles, pattern becomes more complex. Most species spent at least 1 year as larvae in the soil and can live for several years (Thiele, 1977), some also showing so‐called repetitive diapause (similar to a “seed bank” in the soil). Most Carabid beetles are omnivorous although many species have a preference for animal food (Tischler, 1965). Plant residuals in plots previously grown with WR lead to an increase in springtails and in our plots larvae in the soil are not negatively affected by deep ploughing. This can explain that activity density of this group was comparatively high in WR and in plots that were grown with WR in the previous year. However, compared to diplopods, this group is generally considered very mobile—small species are even able to fly. In addition, some species also hibernate as adults. Therefore, they usually move to noncrop areas as field margins and spread to the crop in the subsequent year (Tscharntke, Rand, & Bianchi, 2005). This might explain why the memory effect was not that clear in this group compared with the diplopods.

Overall, and across all taxa considered, we expect that specialist and immobile species are more strongly (usually negatively) affected by crop rotations—as for example rootworm larvae (Esser et al., 2015) or nematodes—this is the reason why crop rotations are applied. Generalist species with a high spatial mobility (species with flying availability) may be less affected by crop rotations although, for sure, crop composition on a landscape scale is very important. Overall, long‐term effects of the preceding crop(s) may be more important than previously thought.

Apart from memory effects, our study also clearly demonstrates that WR has a positive influence on most species groups compared with SB or SM. We can thus conclude that on a landscape scale, a higher percentage of WR may provide more suitable temporary “stepping stone” habitats for insects (as long as insecticide application is limited).

With respect to temporal diversity of crop rotations, the conclusions to be drawn from our study are mixed—mainly because crop identity and diversity were not strictly separated in our design (the design was wheat‐based). Thus, temporal crop diversity effects were likely sampling effects caused by diverse rotations having a higher probability to contain “beneficial” crops. Hence, future studies should aim at disentangling crop identity from crop diversity effects. Yet, for “true” intensively managed farming systems, disentangling identity and diversity may be almost impossible due to self‐incompatibility of crop such as WR, meaning that many crops cannot be grown in a continuously for many years in a row (Aigner & Wendland, 2014).

## 5 CONCLUSIONS AND OUTLOOK

In this study, both crop identity and preceding crops influenced species composition and activity density of aboveground arthropods. Of course, results from a crop rotation experiment such as the present study cannot easily be scaled up to farm or landscape level. Yet, there is compelling evidence that cropping system diversification in general has strong effects on arthropods also on larger spatial scales and in real‐world landscapes (Lichtenberg et al., 2017). Temporal cropping system diversification can only be one of many tools to diversify farming systems, and care needs to be taken that rotations contain “beneficial” crops that enhance arthropod diversity. It is an erroneous belief that a temporal variation of grains within a rotation leads to an increase in insect diversity because wheat, rye, and barley have a similar vegetation structure and a similar microclimate and will therefore also provide habitat for the same species. Including WR (or other taxa) as dicotyledonous flowering crop with dense vegetation structure, for example, might be a helpful tool to increase insect abundance, richness, or biomass and provide habitat for other insect species. For sure, taking into consideration that field sites in the first instance serve for food production or the production of energy crops, it is also necessary to include current market situation to evaluate which crop should be grown on the field.

To conserve landscape‐wide arthropod biodiversity, two prerequisites are necessary: (a) arthropod biodiversity monitoring needs to be done also in intensively managed arable fields, and (b) temporal and spatial diversity of crop fields, and crop species identity, need to be designed to become more “arthropod‐friendly.” Although most species to be found in arable fields are generally considered common and ubiquitous, they form important components of ecological networks and may serve as food source for higher trophic levels (e.g., small game such as pheasants or partridges) or provide ecosystem services such as pollination or pest control.

From a landscape perspective, crop rotations create an annually changing mosaic pattern that provides habitat for a varying amount of species. Most species have to redistribute themselves depending on cropping patterns in the landscape (Vasseur et al., 2013). Therefore, if we manage to create a system that provides different crops offering a large variety of structural elements, microhabitats, and food sources to animals within 1 year but also integrated over several years, this will help diversifying even intensively managed European farmland on areas where other approaches may fail.

## CONFLICT OF INTEREST

None declared.

## AUTHOR CONTRIBUTION

PG, HJK, and colleagues designed the experiment. MM, DO, and CS developed sampling design, analyzed, and wrote a first draft of the manuscript, MM collected the field data. All authors contributed to writing and discussion of the results.

## Data Availability

The data supporting this study are available at https://doi.org/10.5061/dryad.b30mh0p.
