# Supplementary material for: Crop identity and memory effects on aboveground arthropods in a long‐term crop rotation experiment
Source: Ecol Evol. 2019 May 29;9(12):7307–23. doi: 10.1002/ece3.5302 (PMC6662258; doi:10.1002/ece3.5302)
Supplement: Supplementary file 1 [file ECE3-9-7307-s001.docx]

**Tables**

**Table S1**: Mean activity density and species richness of carabids, spiders and iso- and diplopods in response to crop identity (in 2016, 2015 and 2014). Highest values highlighted in grey. Values are predictions from generalized linear mixed-effects models. CI, confidence interval. Crop abbreviations: GP = grain pea, SB = sugar beet, SM = silage maize, WR = winter oilseed rape, WW = winter wheat

|  | | | **Activity density** | | | **Species richness** | | |
| --- | --- | --- | --- | --- | --- | --- | --- | --- |
| **Taxon** | **Year** | **Crop identity** | **Mean** | **Lower CI** | **Upper CI** | **Mean** | **Lower CI** | **Upper CI** |
| **Carabids** | **2016** | **GP** | 176.5 | 122.4 | 254.4 | 3.6 | 2.7 | 5.0 |
|  |  | **SB** | 96.3 | 77.3 | 120.1 | 3.1 | 2.6 | 3.7 |
|  |  | **SM** | 118.6 | 95.5 | 147.4 | 3.7 | 3.1 | 4.5 |
|  |  | **WR** | 284.2 | 220.6 | 366.2 | 4.4 | 3.5 | 5.5 |
|  |  | **WW** | 116.2 | 103.7 | 130.1 | 4.8 | 4.3 | 5.3 |
|  | **2015** | **GP** | 102.6 | 64.2 | 163.9 | 2.9 | 2.1 | 4.1 |
|  |  | **SB** | 106.0 | 80.9 | 138.9 | 5.0 | 4.1 | 6.1 |
|  |  | **SM** | 87.8 | 67.0 | 115.0 | 4.3 | 3.6 | 5.2 |
|  |  | **WR** | 142.4 | 102.3 | 198.3 | 5.3 | 4.2 | 6.6 |
|  |  | **WW** | 141.8 | 123.1 | 163.3 | 4.0 | 3.6 | 4.4 |
|  | **2014** | **GP** | 127.3 | 77.7 | 208.6 | 4.4 | 3.1 | 6.3 |
|  |  | **SB** | 156.8 | 117.9 | 208.6 | 4.0 | 3.3 | 4.9 |
|  |  | **SM** | 97.6 | 73.4 | 129.8 | 4.8 | 3.9 | 5.8 |
|  |  | **WR** | 124.2 | 87.6 | 176.1 | 4.9 | 3.8 | 6.3 |
|  |  | **WW** | 124.5 | 107.2 | 144.4 | 4.1 | 3.6 | 4.5 |
| **Spiders** | **2016** | **GP** | 85.7 | 60.6 | 121.1 | 7.3 | 6.0 | 9.0 |
|  |  | **SB** | 105.1 | 87.8 | 125.9 | 7.6 | 6.8 | 8.6 |
|  |  | **SM** | 92.7 | 76.5 | 112.3 | 8.7 | 7.8 | 9.9 |
|  |  | **WR** | 109.7 | 88.4 | 136.1 | 8.4 | 7.2 | 9.7 |
|  |  | **WW** | 110.5 | 100.8 | 121.1 | 7.9 | 7.4 | 8.4 |
|  | **2015** | **GP** | 129.3 | 98.8 | 169.4 | 7.3 | 5.9 | 9.1 |
|  |  | **SB** | 120.1 | 102.2 | 141.2 | 9.1 | 8.2 | 10.2 |
|  |  | **SM** | 101.6 | 85.2 | 121.1 | 8.4 | 7.5 | 9.5 |
|  |  | **WR** | 117.7 | 96.3 | 143.7 | 7.0 | 6.0 | 8.2 |
|  |  | **WW** | 98.5 | 89.7 | 108.1 | 7.9 | 7.5 | 8.4 |
|  | **2014** | **GP** | 97.2 | 69.2 | 136.5 | 8.6 | 7.1 | 10.5 |
|  |  | **SB** | 109.1 | 90.7 | 131.2 | 8.6 | 7.7 | 9.6 |
|  |  | **SM** | 124.5 | 104.6 | 148.0 | 8.6 | 7.7 | 9.6 |
|  |  | **WR** | 116.0 | 93.1 | 144.6 | 6.6 | 5.8 | 7.6 |
|  |  | **WW** | 105.4 | 95.6 | 116.4 | 7.8 | 7.3 | 8.3 |
| **Iso-/Diplopods** | **2016** | **GP** | 7.7 | 3.2 | 18.5 | 2.1 | 1.4 | 3.1 |
|  |  | **SB** | 9.6 | 5.8 | 15.9 | 2.3 | 1.8 | 2.8 |
|  |  | **SM** | 11.7 | 7.0 | 19.3 | 2.0 | 1.6 | 2.6 |
|  |  | **WR** | 5.6 | 3.0 | 10.4 | 1.5 | 1.1 | 2.1 |
|  |  | **WW** | 9.7 | 7.5 | 12.7 | 2.2 | 2.0 | 2.5 |
|  | **2015** | **GP** | 14.2 | 6.2 | 32.9 | 2.7 | 1.9 | 3.8 |
|  |  | **SB** | 5.2 | 3.2 | 8.4 | 2.1 | 1.7 | 2.6 |
|  |  | **SM** | 7.0 | 4.3 | 11.3 | 1.9 | 1.5 | 2.4 |
|  |  | **WR** | 22.4 | 12.4 | 40.6 | 2.2 | 1.7 | 2.8 |
|  |  | **WW** | 9.2 | 7.1 | 11.8 | 2.1 | 1.8 | 2.3 |
|  | **2014** | **GP** | 4.2 | 1.9 | 9.4 | 2.0 | 1.4 | 2.9 |
|  |  | **SB** | 6.8 | 4.3 | 10.8 | 1.8 | 1.4 | 2.2 |
|  |  | **SM** | 6.1 | 3.9 | 9.7 | 2.1 | 1.7 | 2.6 |
|  |  | **WR** | 16.2 | 9.2 | 28.6 | 2.7 | 2.1 | 3.4 |
|  |  | **WW** | 11.3 | 8.9 | 14.4 | 2.1 | 1.8 | 2.3 |

**Table S2**: Multiple comparisons (Tukey’s all-pairwise comparisons) of the effects of crop identity in 2016, 2015 and 2014 on activity density. Significant differences are highlighted in bold. Std. Error, 1 standard error of the mean. Crop abbreviations: GP = grain pea, SB = sugar beet, SM = silage maize, WR = winter oilseed rape, WW = winter wheat

| Individuals | | 2016 | | | | 2015 | | | | 2014 | | | |
| --- | --- | --- | --- | --- | --- | --- | --- | --- | --- | --- | --- | --- | --- |
|  |  | Estimate | Std. Error | z value | Pr(>\|z\|) | Estimate | Std. Error | z value | Pr(>\|z\|) | Estimate | Std. Error | z value | Pr(>\|z\|) |
| Carabids | SB - GP | **-0.596** | **0.210** | **-2.836** | **0.035** | 0.036 | 0.360 | 0.099 | 1.000 | 0.343 | 0.329 | 1.043 | 0.824 |
|  | SM - GP | -0.359 | 0.202 | -1.777 | 0.373 | -0.137 | 0.368 | -0.373 | 0.996 | -0.230 | 0.353 | -0.651 | 0.964 |
|  | WR - GP | 0.506 | 0.189 | 2.670 | 0.055 | 0.307 | 0.366 | 0.837 | 0.912 | 0.017 | 0.361 | 0.046 | 1.000 |
|  | WW - GP | -0.410 | 0.177 | -2.314 | 0.133 | 0.3981 | 0.323 | 1.234 | 0.715 | 0.060 | 0.308 | 0.192 | 1.000 |
|  | SM - SB | 0.238 | 0.173 | 1.377 | 0.629 | -0.173 | 0.263 | -0.658 | 0.962 | -0.573 | 0.240 | -2.390 | 0.109 |
|  | WR - SB | **110.196** | **0.158** | **6.972** | **<0.001** | 0.271 | 0.260 | 1.041 | 0.824 | -0.327 | 0.253 | -1.293 | 0.679 |
|  | WW - SB | 0.187 | 0.143 | 1.306 | 0.675 | 0.363 | 0.194 | 1.870 | 0.315 | -0.284 | 0.168 | -1.690 | 0.421 |
|  | WR - SM | **0.864** | **0.146** | **5.900** | **<0.001** | 0.444 | 0.271 | 1.636 | 0.454 | 0.246 | 0.282 | 0.871 | 0.9 |
|  | WW - SM | -0.051 | 0.130 | -0.393 | 0.995 | 0.535 | 0.209 | 2.567 | 0.070 | 0.289 | 0.210 | 1.374 | 0.627 |
|  | WW - WR | **-0.915** | **0.110** | **-8.327** | **<0.001** | 0.092 | 0.205 | 0.446 | 0.991 | 0.043 | 0.225 | 0.19 | 1.000 |
| Spiders | SB - GP | 0.205 | 0.195 | 1.051 | 0.82 | -0.074 | 0.157 | -0.471 | 0.989 | 0.071 | 0.184 | 0.386 | 0.995 |
|  | SM - GP | 0.079 | 0.197 | 0.398 | 0.994 | -0.242 | 0.161 | -1.505 | 0.545 | 0.223 | 0.181 | 1.232 | 0.718 |
|  | WR - GP | 0.247 | 0.203 | 1.214 | 0.728 | -0.095 | 0.168 | -0.564 | 0.979 | 0.168 | 0.192 | 0.876 | 0.899 |
|  | WW - GP | 0.254 | 0.179 | 1.424 | 0.593 | -0.272 | 0.142 | -1.911 | 0.298 | 0.053 | 0.168 | 0.316 | 0.998 |
|  | SM - SB | -0.126 | 0.131 | -0.959 | 0.864 | -0.168 | 0.119 | -1.408 | 0.608 | 0.152 | 0.122 | 1.243 | 0.711 |
|  | WR - SB | 0.042 | 0.140 | 0.302 | 0.998 | -0.021 | 0.128 | -0.16 | 1.000 | 0.097 | 0.138 | 0.705 | 0.952 |
|  | WW - SB | 0.050 | 0.101 | 0.491 | 0.987 | -0.198 | 0.093 | -2.129 | 0.197 | -0.018 | 0.101 | -0.18 | 1.000 |
|  | WR - SM | 0.168 | 0.144 | 1.168 | 0.755 | 0.147 | 0.133 | 1.108 | 0.793 | -0.055 | 0.134 | -0.411 | 0.993 |
|  | WW - SM | 0.176 | 0.106 | 1.654 | 0.443 | -0.030 | 0.099 | -0.306 | 0.998 | -0.170 | 0.096 | -1.781 | 0.367 |
|  | WW - WR | 0.007 | 0.117 | 0.061 | 1.000 | -0.178 | 0.110 | -1.613 | 0.474 | -0.115 | 0.115 | -1.004 | 0.844 |
| Iso-/Diplopods | SB - GP | 0.238 | 0.663 | 0.36 | 0.996 | -0.840 | 0.570 | -1.473 | 0.562 | 5.26E+02 | 8.25E+02 | 0.638 | 0.965 |
|  | SM - GP | 0.563 | 0.643 | 0.876 | 0.896 | -0.488 | 0.532 | -0.918 | 0.883 | 5.26E+02 | 8.25E+02 | 0.638 | 0.965 |
|  | WR - GP | -0.288 | 0.761 | -0.378 | 0.995 | 0.573 | 0.485 | 1.180 | 0.749 | 1.59E+03 | 7.92E+02 | 2.003 | 0.239 |
|  | WW - GP | 0.440 | 0.607 | 0.725 | 0.945 | -0.173 | 0.451 | -0.384 | 0.995 | 1.20E+03 | 7.64E+02 | 1.565 | 0.489 |
|  | SM - SB | 0.325 | 0.397 | 0.819 | 0.917 | 0.351 | 0.491 | 0.715 | 0.95 | -8.02E-13 | 4.73E+02 | 0 | 1.000 |
|  | WR - SB | -0.526 | 0.568 | -0.926 | 0.876 | **14.123** | **0.440** | **3.211** | **0.011** | 1.06E+03 | 4.13E+02 | 2.569 | 0.066 |
|  | WW - SB | 0.201 | 0.334 | 0.603 | 0.972 | 0.667 | 0.402 | 1.658 | 0.442 | 6.70E+02 | 3.57E+02 | 1.876 | 0.303 |
|  | WR - SM | -0.851 | 0.546 | -1.560 | 0.498 | **10.609** | **0.389** | **2.726** | **0.047** | 1.06E+03 | 4.13E+02 | 2.569 | 0.067 |
|  | WW - SM | -0.123 | 0.294 | -0.42 | 0.993 | 0.315 | 0.346 | 0.911 | 0.885 | 6.70E+02 | 3.57E+02 | 1.876 | 0.302 |
|  | WW - WR | 0.728 | 0.502 | 1.449 | 0.571 | **-0.746** | **0.268** | **-2.787** | **0.039** | -3.90E+02 | 2.72E+02 | -1.435 | 0.576 |

**Table S3**: Multiple comparisons (Tukey’s all-pairwise comparisons) of the effects of crop identity in 2016, 2015 and 2014 on species numbers. Significant differences are highlighted in bold. Std. Error, 1 standard error of the mean. Crop abbreviations: GP = grain pea, SB = sugar beet, SM = silage maize, WR = winter oilseed rape, WW = winter wheat

| Species  Richness | | 2016 | | | | 2015 | | | | 2014 | | | |
| --- | --- | --- | --- | --- | --- | --- | --- | --- | --- | --- | --- | --- | --- |
|  |  | Estimate | Std. Error | z value | Pr(>\|z\|) | Estimate | Std. Error | z value | Pr(>\|z\|) | Estimate | Std. Error | z value | Pr(>\|z\|) |
| Carabids | SB - GP | -0.164 | -0.201 | 0.818 | 0.919 | 0.554 | 0.218 | 2.546 | 0.074 | -1.27E+02 | 2.01E+02 | -0.631 | 0.968 |
|  | SM - GP | 0.087 | 0.195 | 0.447 | 0.991 | 0.418 | 0.220 | 1.897 | 0.300 | 6.90E+01 | 1.96E+02 | 0.352 | 0.996 |
|  | WR - GP | 0.205 | 0.202 | 1.014 | 0.838 | 0.575 | 0.226 | 2.548 | 0.073 | 6.90E+01 | 2.07E+02 | 0.333 | 0.997 |
|  | WW - GP | 0.298 | 0.176 | 1.694 | 0.418 | 0.318 | 0.206 | 1.542 | 0.514 | -1.03E+02 | 1.80E+02 | -0.571 | 0.978 |
|  | SM - SB | 0.251 | 0.142 | 1.767 | 0.374 | -0.137 | 0.128 | -1.068 | 0.811 | 1.96E+02 | 1.42E+02 | 1.378 | 0.627 |
|  | WR - SB | 0.369 | 0.152 | 2.424 | 0.101 | 0.021 | 0.137 | 0.154 | 0.999 | 1.96E+02 | 1.57E+02 | 1.245 | 0.712 |
|  | WW - SB | **0.462** | **0.116** | **4.002** | **<0.001** | -0.237 | 0.101 | -2.339 | 0.122 | 2.43E+01 | 1.18E+02 | 0.205 | 1.000 |
|  | WR - SM | 0.118 | 0.144 | 0.819 | 0.918 | 0.158 | 0.141 | 1.117 | 0.784 | 5.59E-14 | 1.51E+02 | 0 | 1.000 |
|  | WW - SM | 0.211 | 0.104 | 2.029 | 0.237 | -0.100 | 0.107 | -0.94 | 0.872 | -1.72E+02 | 1.10E+02 | -1.562 | 0.506 |
|  | WW - WR | 0.093 | 0.117 | 0.794 | 0.927 | -0.258 | 0.118 | -2.193 | 0.169 | -1.72E+02 | 1.29E+02 | -1.330 | 0.658 |
| Spiders | SB - GP | 0.044 | 0.125 | 0.355 | 0.996 | 0.217 | 0.120 | 1.810 | 0.349 | 1.27E+01 | 1.11E+02 | 0.115 | 1.000 |
|  | SM - GP | 0.192 | 0.123 | 1.562 | 0.503 | 0.141 | 0.121 | 1.167 | 0.756 | -9.89E-14 | 1.11E+02 | 0 | 1.000 |
|  | WR - GP | 0.148 | 0.130 | 1.132 | 0.777 | -0.047 | 0.131 | -0.354 | 0.996 | -2.62E+02 | 1.24E+02 | -2.117 | 0.200 |
|  | WW - GP | 0.079 | 0.114 | 0.699 | 0.953 | 0.079 | 0.111 | 0.716 | 0.949 | -9.15E+01 | 1.01E+02 | -0.904 | 0.888 |
|  | SM - SB | 0.148 | 0.084 | 1.760 | 0.379 | -0.076 | 0.080 | -0.956 | 0.866 | -1.27E+01 | 7.85E+01 | -0.162 | 0.999 |
|  | WR - SB | 0.103 | 0.094 | 1.093 | 0.799 | **-0.264** | **0.095** | **-2.782** | **0.040** | **-2.75E+02** | **9.55E+01** | **-2.881** | **0.03** |
|  | WW - SB | 0.035 | 0.069 | 0.505 | 0.986 | -0.138 | 0.063 | -2.178 | 0.175 | -1.04E+02 | 6.32E+01 | -1.649 | 0.448 |
|  | WR - SM | -0.045 | -0.092 | 0.488 | 0.987 | -0.188 | 0.096 | -1.954 | 0.273 | **-2.62E+02** | **9.57E+01** | **-2.742** | **0.044** |
|  | WW - SM | -0.113 | -0.065 | 1.729 | 0.398 | -0.062 | 0.065 | -0.948 | 0.869 | -9.15E+01 | 6.35E+01 | -1.441 | 0.584 |
|  | WW - WR | -0.068 | -0.078 | 0.872 | 0.901 | 0.126 | 0.083 | 1.517 | 0.532 | 1.71E+02 | 8.36E+01 | 2.045 | 0.231 |
| Iso-/Diplopods | SB - GP | 1.05E+02 | 2.20E+02 | 0.48 | 0.988 | -0.234 | 0.205 | -1.142 | 0.773 | -0.118 | 0.225 | -0.524 | 0.984 |
|  | SM - GP | -5.14E-13 | 2.22E+02 | 0 | 1.000 | -0.345 | 0.208 | -1.658 | 0.446 | 0.054 | 0.220 | 0.246 | 0.999 |
|  | WR - GP | -2.88E+02 | 2.49E+02 | -1.157 | 0.761 | -0.208 | 0.218 | -0.952 | 0.869 | 0.288 | 0.225 | 1.279 | 0.690 |
|  | WW - GP | 8.70E+01 | 2.00E+02 | 0.434 | 0.992 | -0.258 | 0.181 | -1.422 | 0.599 | 0.030 | 0.200 | 0.149 | 0.999 |
|  | SM - SB | -1.05E+02 | 1.53E+02 | -0.687 | 0.956 | -0.111 | 0.162 | -0.687 | 0.957 | 0.172 | 0.159 | 1.078 | 0.807 |
|  | WR - SB | -3.93E+02 | 1.89E+02 | -2.076 | 0.216 | 0.026 | 0.175 | 0.149 | 1.000 | 0.405 | 0.166 | 2.441 | 0.098 |
|  | WW - SB | -1.84E+01 | 1.19E+02 | -0.154 | 1.000 | -0.024 | 0.126 | -0.192 | 1.000 | 0.148 | 0.131 | 1.131 | 0.778 |
|  | WR - SM | -2.88E+02 | 1.93E+02 | -1.494 | 0.547 | 0.137 | 0.179 | 0.767 | 0.936 | 0.234 | 0.159 | 1.465 | 0.568 |
|  | WW - SM | 8.70E+01 | 1.24E+02 | 0.7 | 0.953 | 0.087 | 0.132 | 0.661 | 0.962 | -0.024 | 0.122 | -0.199 | 0.999 |
|  | WW - WR | 3.75E+02 | 1.67E+02 | 2.247 | 0.151 | -0.050 | 0.147 | -0.342 | 0.997 | -0.258 | 0.13 | -1.975 | 0.264 |
